# Idiopathic Choroidal Neovascularisation as the Inaugural Sign of Multiple Evanescent White Dot Syndrome

**DOI:** 10.4103/0974-9233.65490

**Published:** 2010

**Authors:** Marina Papadia, Carl P Herbort

**Affiliations:** 1 Centre for Specialized Ophthalmic Care (COS), University of Genova, Genova, Italy; 2 Eye Clinic, Department of Neurosciences, Ophthalmology and Genetics, University of Genova, Genova, Italy; 3 University of Lausanne, Lausanne, Switzerland

**Keywords:** Idiopathic Choroidal Neovascularization, Indocyanine Green Angiography, Multiple Evanescent White Dot Syndrome

## Abstract

We report a case of multiple evanescent white dot syndrome (MEWDS) that presented with putative idiopathic choroidal neovascularisation (ICNV) before showing angiographic signs typical of MEWDS. A 16-year-old male presented with unilateral metamorphopsias and visual loss in his left eye. ICNV with subretinal hemorrhage was diagnosed and treated with intravitreal Avastin^®^. Fifteen days later, regression of choriodal neovascularization (CNV) was documented together with the appearance of fluorescein angiography (FA) and indocyanine green angiography (ICGA) signs typical for MEWDS, that included faint mottled FA hyperfluorescence in the mid-peripheral fundus, irregularly shaped mid-peripheral ICGA dark areas in the intermediate angiographic phase that were clearly delineated in the late phase as well as peripapillary hypofluorescence. Fundus examination appeared completely normal during the follow-up except for the CNV hemorrhage noted at the initial visit. This case demonstrates the need to consider ICNV as a diagnosis of exclusion until inflammatory causes have been eliminated. In this case, the underlying occult inflammatory condition would have been missed without the ICGA data that clearly showed signs of MEWDS that was supported by FA findings.

## INTRODUCTION

Choroidal neovascularization (CNV) is characterized by vision loss due to intraretinal exudation or subretinal fluid, hemorrhage, or fibrosis. It results from perturbation of the retinal pigment epithelium (RPE)-Bruch’s membrane complex that is usually related to the aging process. In younger patients, however, CNV can be secondary to myopia, angioid streaks, multifocal choroiditis (MC), inflammatory origin, scars of any origin^1^ and trauma.^2^ In young patients, CNV can occur also in the absence of any apparent primary ocular or systemic disease, and these are generally categorized as idiopathic CNV.^3^ Recently, some authors have demonstrated that idiopathic choroidal neovascularization (CNV) that occurs in younger patients is often inflammatory in nature and sometimes responds to systemic or periocular steroids.^4^

## CASE REPORT

**Figure 1:**
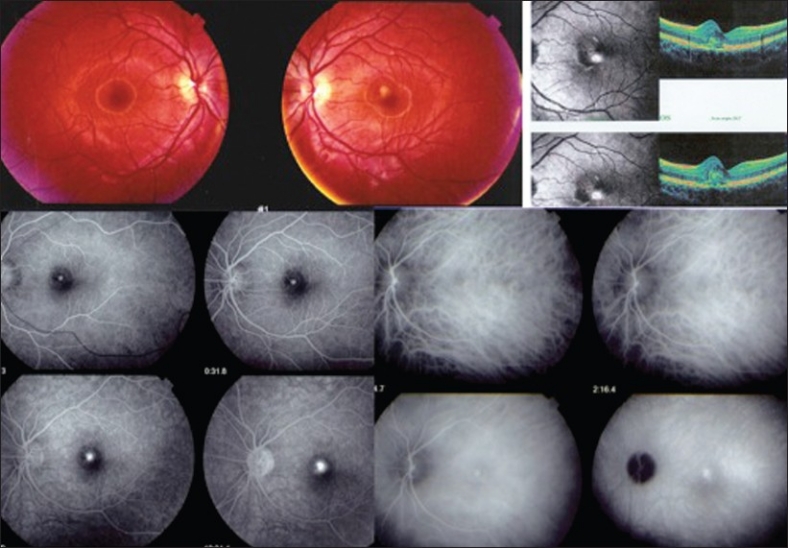
Fundus findings at the first visit. Note the juxtafoveal yellowish lesion in the fundus photograph of the left eye. Optical coherence tomography (OCT) revealed a dome-shaped subretinal lesion along with changes in the retinal pigment epithelium (RPE), which was disrupted and the detachment of neurosensory retina with minimal intraretinal fluid accumulation. On fluorescein angiography (FA), a juxtafoveal hyperfluorescent spot with some leakage in the late phase was noted and first interpreted as the leaking point of a central serous choroidopathy (CSC). Indocyanine green angiography (ICGA) confirmed the presence of a well-defined spot of hyperfluorescence with late phase leakage

We report a case of a 16-year-old man who presented to the Centre for Ophthalmic Specialized Care (COS) in Lausanne, Switzerland with decreased vision in the left eye and metamorphopsias. At presentation, the patient’s best-corrected visual acuity (BCVA) was 20/20 in the right eye (RE) and 20/40 in the left eye (LE), and the intraocular pressure (IOP) was 12 mmHg in both eyes. Slit lamp examination of the anterior segment was normal, without signs of inflammation as measured by laser flare photometry (3.0 ph/ms in the LE) (Kowa FM-500, Kowa Company, Ltd., Electronics and Optics Division, Tokyo, Japan). Vitreous cells were absent. Examination of the fundus in the right eye was normal. The LE showed a juxtafoveal yellowish lesion. OCT (OTI-Spectral OCT/SLO; OTI Inc, Toronto, Canada) confirmed the presence of a dome-shaped subretinal lesion with RPE, disruption, neurosensory retinal detachment with minimal intraretinal fluid accumulation. Fluorescein angiography (FA) showed a juxtafoveal hyperfluorescent spot with some leakage in the late phase of angiography. Indocyanine green angiography (ICGA) showed a well-defined spot of hyperfluorescence with leakage in the late phase [Figure 1]. At this point, a putative diagnosis of central serous choroidopathy was maintained and the previously instituted systemic therapy with 250 mg acetazolamide daily was continued.

**Figure 2:**
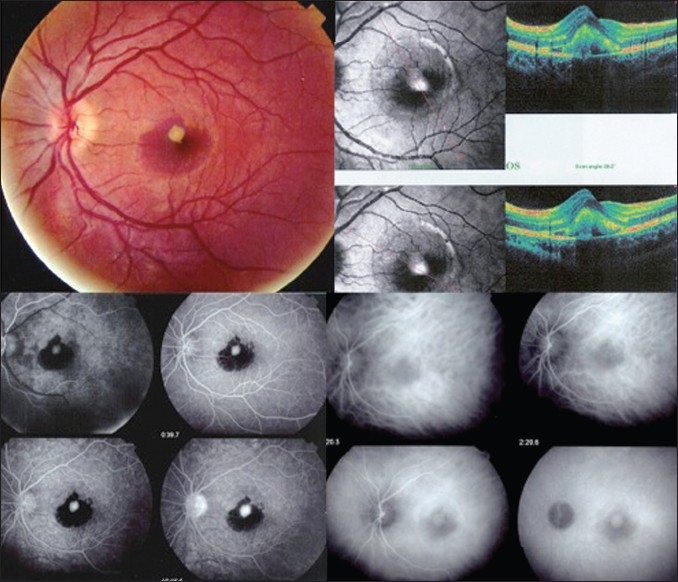
Fundus findings 14 days after the first visit. Note the subretinal hemorrhage on optical coherence tomography (OCT), a prominent dome-shaped lesion above the retinal pigment epithelium (RPE) level likely corresponding to choroidal neovascularization (CNV) with sub- and intraretinal fluid was noted. Flourescein angiography showed the masking effect of the subretinal hemorrhage and the juxtafoveal hyperfluorescent dot representing the CNV and indocyanine green angiography (ICGA) showed a late-phase hyperfluorescent area, corresponding to the CNV.

Fourteen days later, BCVA declined to 20/200 in the LE and a subretinal hemorrhage was present. OCT showed an enlargement of the dome-shaped lesion with an even more prominent thickening of the retina due to enhanced accumulation of intra- and subretinal fluid and the presence of hyperreflective elevation above the RPE level. FA and ICGA confirmed the diagnosis of idiopathic CNV [Figure 2] and the patient received an intravitreal injection of 2 mg of Bevacizumab (Avastin^®^, Genentech, CA).

**Figure 3:**
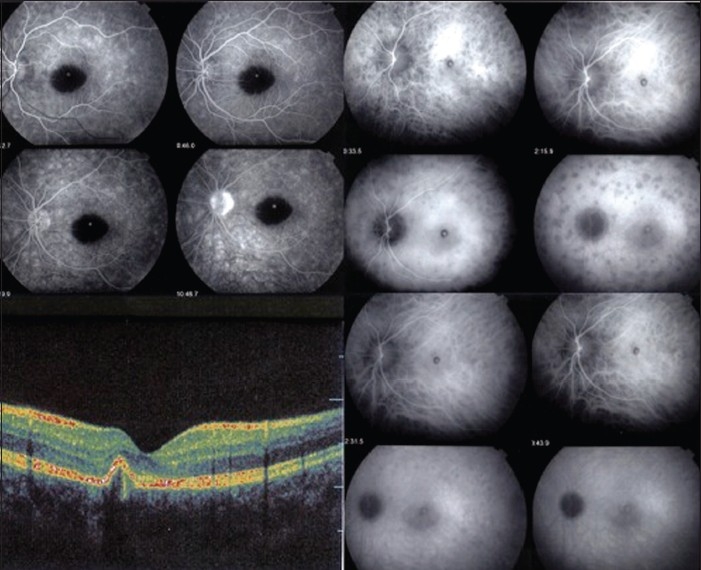
Fundus findings 14 days after intravitreal bevacizumab injection. Flourescein angiography showing regression of choroidal neovascularization (CNV) as well as faint motteled hyperfluorescent areas along the arcades, superiorly visible on early frames (top left) and more diffuse on later frames. ICGA showing regression of CNV. Typical features of multiple evanescent white dot syndrome (MEWDS) including hypofluorescent dark areas best visible on late frames as well as a peripapillary hypofluorescent ring are clearly visible (top right). Indocyanine green angiography (ICGA) after 2 months of systemic corticosteroid therapy showing scarring of CNV and complete resolution of MEWDS ICGA signs (bottom right) and optical coherence tomography (OCT) showing an almost complete resolution of the macular lesion.

Two weeks after the bevacizumab injection, BCVA improved to 20/40 in the LE. The anterior segment was within normal limits without signs of inflammation. Fundus examination appeared normal except for the unchanged appearance of CNV and the hemorrhage. Visual field examination showed a central relative scotoma and the presence of diffuse relative scotomas in the midperiphery. Microperimetry demonstrated the presence of absolute defects with a visual score of 192.0/560 (6.9 dB) (normal range 13–19 dB) (Pattern Polar 3–11°, Size Goldmann III, OTI-Spectral OCT/SLO microperimetry; OTI Inc., Toronto, ON, Canada). A repeat OCT scan confirmed a decrease in elevation of the RPE with the presence of some intraretinal fluid. On FA early, patchy hyperfluorescence was seen mostly along the arcades and hyperfluorescence of CNV decreased. Some of these hyperfluorescent areas were already present in the early phase of the angiogram; others appeared only in the late phase [Figure 3]. Hyperfluorescence of the disk was evident in the late phase. On ICGA, numerous small to mid-size hypofluorescent lesions corresponding to the patchy hyperfluorescence seen on FA were identified in the mid phase but was more prominent in the late phase of the angiogram. Proximal to the optic disk, a halo of hypofluorescence appeared in the intermediate phase and was clearly identified in the late phase of the angiogram [Figure 3]. All these features are typical findings described in MEWDS.^5^ Electroretinography (ERG) was not performed.

At this point, systemic corticosteroid therapy (prednisone 100 mg/daily for 3 days, then 50 mg/daily tapered over 2 months) was initiated due to the inflammatory nature of the CNV. Seven days after initiating systemic corticosteroid therapy, the BCVA improved to 20/25 in the affected eye. The test score of microperimetry increased to 330.0/560 (11.8 dB), with near complete resolution of the central scotoma. On FA, there was reduced hyperfluorescence of the disk in the late phase. ICGA findings were also regressing however, the hypofluorescent dots were still visible in the late phases of the angiograms (not shown).

Two months after systemic corticosteroid therapy, the ocular examination remained stable, with a BCVA in the RE of 20/25. Examination revealed a macular scar and the remaining fundus examination was normal. ICGA and FA showed disappearance of MEWDS lesions [Figure 3]. The corticosteroid therapy was stopped.

Eighteen months after initial presentation, BCVA remained 20/25 in the RE and microperimetry confirmed the presence of a small central scotoma [test score 456.0/560 (16.3 dB)]. The visual field defects completely resolved. The OCT scan revealed further reduction of the RPE elevation [Figure 3].

## DISCUSSION

ICNV that occurs in patients under 50 of age usually has an underlying inflammatory cause. Giovannini *et al*. suggested a link between ICNV and inflammatory choroidopathies based on common ICGA findings in both conditions consisting mostly of hypofluorescent areas and alterations in choroidal permeability.^6^ These authors further indicated that some cases of ICNV could evolve into inflammatory chorioretinopathies.^6^ Callanan and Gass also suggested that ICNV was likely secondary to choroidal inflammation.^7^

MEWDS is an inflammatory choriocapillaropathy which is mostly unilateral^8^,^9^ and is characterized by the presence of multiple faint whitish lesions in the midperipheral fundus. The presence of the white dots transiently affects the function of photoreceptors and RPE.^10^ The cause of this dysfunction is related to disturbances in choriocapillaris perfusion in inflammatory choriocapillaropathies.^11^ It has been postulated that an outer retinal ischemia caused choriocapillaris nonperfusion resulting in photoreceptor dysfunction.^12^ MEWDS is rarely complicated by CNV because in contrast to MC, ischemia is limited in extent and duration.^13^ Nevertheless any ischemia at the level of the choriocapillaris–RPE complex can trigger neovascularization, and there are several reports in the literature on CNV complicating MEWDS.^8^,^14^–^17^ In the past, ERG was performed to aid in the diagnosis of MEWDS. However with the availability of newer more precise methods such as ICGA, reports on ERG in MEWDS are rare and contradictions exist on the interpretation of ERG findings in MEWDS.^10^,^13^,^18^

The case presented here is particularly interesting since the underlying inflammatory condition of MEWDS at the origin of ICNV would have been missed without the dual angiographic workup including FA and ICGA. Additionally, the CNV preceded the angiographic signs of MEWDS indicating a silent phase during which occult inflammatory events occurred. These triggers can induce both CNV and inflammatory changes at the level of the choriocapillaris. A similar constellation of signs was previously described by Machida *et al*.^19^ who reported two cases compatible with MEWDS that surprisingly developed ICNV in the contralateral eye with concomitant signs of MEWDS in one case and preceding the inflammatory choriocapillaropathy in the other case.

MEWDS is unilateral in more than 90%^20^ of cases with the exception of the two cases reported by Machida *et al*.^19^ which indicates that the contralateral eye could be involved subclinically more often than previously reported. These findings in our case are akin to the classic picture of MEWDS respecting unilaterality with ICNV and MEWDS presenting in the same eye.

Inflammatory events alone at the level of the choriocapillaris–RPE complex may trigger the development of CNV. One factor that possibly increases the risk for CNV is ischemia at the level of the RPE and outer retina caused by choriocapillaris nonperfusion. In MC, ICGA analysis of the choriocapillaris has shown that in addition to the apparent foci, there is extensive occult choriocapillaris nonperfusion explaining the high rate of CNV in MC.^21^

The systemic corticosteroid administered in this case once the inflammatory nature of the CNV was identified, likely helped with further regression of the CNV following the initial response obtained by the intravitreal Avastin^®^ injection. The corticosteroid treatment likely also hastened the resolution of MEWDS. Eighteen months after the acute episode, there was no sign of recurrence of the inflammation or the CNV, corresponding to the usual behavior of MEWDS, a unilateral, nonrecurrent disease.
